# The Arabidopsis GPR1 Gene Negatively Affects Pollen Germination, Pollen Tube Growth, and Gametophyte Senescence

**DOI:** 10.3390/ijms18061303

**Published:** 2017-06-21

**Authors:** Xiao Yang, Qinying Zhang, Kun Zhao, Qiong Luo, Shuguang Bao, Huabin Liu, Shuzhen Men

**Affiliations:** Department of Plant Biology and Ecology, College of Life Sciences, Nankai University, Tianjin 300071, China; yang_xiao2016@163.com (X.Y.); zhangqinying@mail.nankai.edu.cn (Q.Z.); aslan1102@163.com (K.Z.); qiongluo2016@mail.nankai.edu.cn (Q.L.); shuguang@mail.nankai.edu.cn (S.B.); liuhuabin@mail.nankai.edu.cn (H.L.)

**Keywords:** pollen germination, pollen tube, gametophyte, senescence, expression, pollen exine

## Abstract

Genes essential for gametophyte development and fertilization have been identified and studied in detail; however, genes that fine-tune these processes are largely unknown. Here, we characterized an unknown Arabidopsis gene, *GTP-BINDING PROTEIN RELATED1* (*GPR1*). *GPR1* is specifically expressed in ovule, pollen, and pollen tube. Enhanced green fluorescent protein-tagged GPR1 localizes to both nucleus and cytoplasm, and it also presents in punctate and ring-like structures. *gpr1* mutants exhibit no defect in gametogenesis and seed setting, except that their pollen grains are pale in color. Scanning electron microscopy analyses revealed a normal patterned but thinner exine on *gpr1* pollen surface. This may explain why *gpr1* pollen grains are pale. We next examined whether *GPR1* mutation affects post gametogenesis processes including pollen germination, pollen tube growth, and ovule senescence. We found that *gpr1* pollen grains germinated earlier, and their pollen tubes elongated faster. Emasculation assay revealed that unfertilized *gpr1* pistil expressed the senescence marker *P_BFN1_:GUS* (GUS: a reporter gene that encodes β-glucuronidase) one-day earlier than the wild type pistil. Consistently, ovules and pollen grains of *gpr1* mutants showed lower viability than those of the wild type at 4 to 5 days post anthesis. Together, these data suggest that GPR1 functions as a negative regulator of pollen germination, pollen tube growth, and gametophyte senescence to fine-tune the fertilization process.

## 1. Introduction

Successful sexual reproduction of flowering plants depends on pollination, pollen tube growth, and double fertilization. Mature pollen, in a partially dehydrated and dormant state, release from anther to adhere to the female stigmatic tissue via wind, animal pollinators or direct contact. If the pollen grain is compatible with the stigma, it will soon hydrate and start to germinate. Previous studies have revealed that pollen germination is a highly controlled process [1,2,3].

In Arabidopsis, only a few genes have been reported to inhibit pollen germination. The *raring-to-go* (*rtg*) mutant was identified for precocious pollen germination within the anther [4]. However, the *RTG* gene has not been cloned and the molecular mechanism involved remains unknown. Precocious pollen germination was also observed in mutants that genetically altered callose synthesis, including *CALLOSE SYNTHASE9* gene knockout mutant *cs9* and *CALLOSE SYNTHASE5* over-expressing line *CALS5*. Both of them displayed abnormal callose deposition during microsporogenesis, and their pollen grains germinated precociously at bicellular stage [5]. In addition, precocious pollen germination also results from a disruption of inositol polyphosphate 5-phosphatase 12 (5PT12), which controls the cellular Ins(1,4,5)*P3*/Ca^2+^ levels. The knockout mutant *5pt12* displays normal pollen development and pollen dehydration, revealing that independent of dehydration, the Ins(1,4,5)*P3*/Ca^2+^ levels is crucial for maintaining pollen dormancy [6]. Very recently, a protein that contains seven WD40 repeats was identified to inhibit pollen germination in moist environments [7]. This protein was named JINGUBANG (JGB). Further analysis revealed that JGB interacts with the transcription factor TCP4 to control pollen jasmonic acid (JA) synthesis [7].

As pollen tube grows, female tissue produces signals that direct the pollen tube to the embryo sac for fertilization [8,9,10,11]. Proper elongation of the growing tube is essential for fertilization. Pollen tube growth is driven by two major biochemical mechanisms: a calcium gradient at the growing tip and the highly dynamic subapical actin cytoskeleton [12,13,14]. Pollen tubes exhibit a steep, tip-focused cytoplasmic Ca^2+^ gradient. In the tip of growing pollen tube, free Ca^2+^ concentration is approximately several micromolar, whereas in the shank of the tube its concentration is approximately 100 nanomolar [15]. As a central regulator of pollen germination and tube growth, calcium also regulates the cytoskeleton via actin binding proteins [16,17,18]. After fertilization, the pistil undergoes growth and differentiation to become a fruit, whereas if ovules are not fertilized within anthesis, the pistil will initiate senescence process [19,20]. Therefore, ovule lifespan is an important factor in determining the ability to set fruits and produce seeds. However, the post-anthesis development of the unfertilized ovules has received little attention.

In order to isolate sperm-cell-expressed transcripts, Xu et al. isolated and purified tobacco sperm cells from in vitro grown pollen tubes, then extracted mRNA from these sperm cells to construct a cDNA library. By screening of this library they identified two sperm-cell-expressed transcripts, and named them *NtS1* and *NtS2*. Further analysis showed that *NtS1* and *NtS2* transcription levels were higher in the pollen than in the sperm cell, suggesting that both genes were not sperm-cell-specific [21]. *NtS1* codes for a protein with a polygalacturonase active site. The putative NtS2 protein shares 65% amino acid sequence similarity to an unknown Arabidopsis protein encoded by the At3g23860 gene [21].

In this study, we characterized the unknown Arabidopsis At3g23860 gene. In Arabidopsis TAIR10, this gene was annotated to encode a GTP-binding related protein (http://www.arabidopsis.org). Therefore, we named it as *GTP-BINDING PROTEIN RELATED1* (*GPR1*). *GPR1* encodes a putative protein of 231 amino acids. BLAST searches identified no recognizable domain in GPR1. We analyzed the expression pattern of the *GPR1* gene using reverse transcription (RT)-polymerase chain reaction (PCR) and *GPR1* promoter:*GUS* (*ProGPR1:GUS*) transgenic plants, and found that *GPR1* gene was specifically expressed in male and female gametophytes and pollen tubes. We obtained two insertion mutant alleles of the *GPR1* gene, named as *gpr1-1* (SALKseq_034266, in Col background) and *gpr1-2* (CSHL_GT24095, in L*er* background). Although *gpr1* mutants were not defective in gametogenesis and fertilization, their pollen grains displayed pale yellow color compared with those of wild type. Scanning electron microscopy examination showed that *gpr1* mutant pollen grains had a thinner exine surface compared with those of wild type, suggesting that *GPR1* plays a role in pollen coat formation. We also performed in vitro and in vivo pollen germination assay, and found that pollen grains of the *gpr1* mutants germinated earlier and their pollen tubes elongated faster. Finally, we performed emasculation and hand pollination analysis, and demonstrated that loss of *GPR1* function resulted in early senescence of ovules and pollen grains.

## 2. Results

### 2.1. GPR1 Is Specifically Expressed in Ovule, Pollen and Pollen Tube

**Figure 1:**
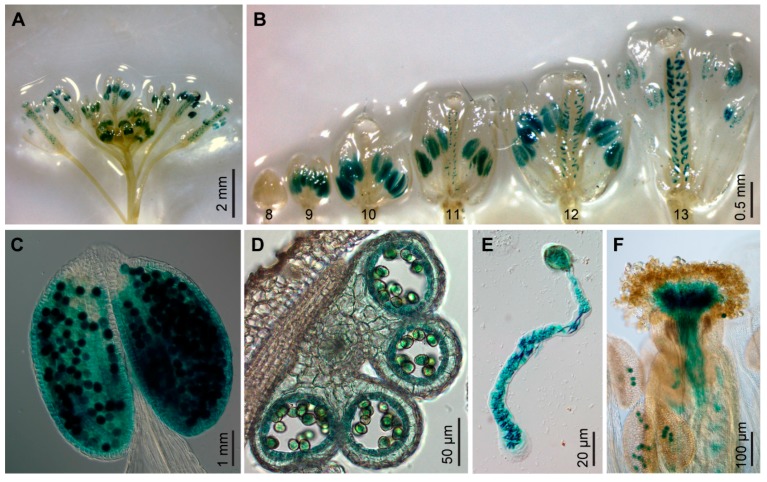
*GPR1* promoter:*GUS* (*ProGPR1:GUS*) is specifically expressed in anthers, ovules and pollen tubes. (**A**) Expression of *ProGPR1:GUS* in inflorescence. (**B**) Flowers at different developmental stages, showing GUS staining in anthers from stage 9 to 13, and in ovules from stage 11 to 13. (**C**) Anther at stage 12, showing GUS signals in pollen grains. (**D**) Cross section of an anther at stage 11, showing GUS signals in tapetum and pollen grains. (**E**,**F**) Pollen grain germinated in vitro (**E**) and on pistil (**F**). Note GUS signals in pollen tube.

**Figure 2:**
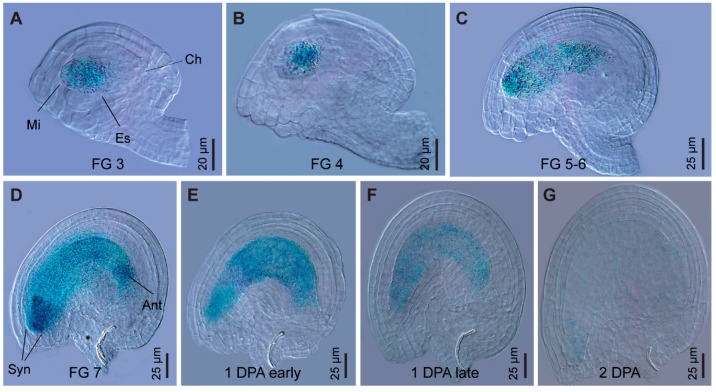
*ProGPR1:GUS* is specifically expressed in embryo sac during female gametophyte development. (**A**–**D**) GUS staining in ovules of *ProGPR1:GUS* plants at female gametophyte (FG) developmental stages FG 3 (**A**), FG 4 (**B**), FG 5-6 (**C**) and FG 7 (**D**). Mi, micropylar pole; Ch, chalazal pole; Es, embryo sac; Syn, synergids; Ant, antipodals. (**E**,**F**) Seeds at 1 day post anthesis (DPA). (**G**) Seed at 2 DPA.

*GPR1* shares high similarity to the tobacco sperm-cell-expressed transcript *NtS2*. Therefore, we investigated whether *GPR1* gene is also specifically expressed in pollen grains. RT-PCR analysis revealed that *GPR1* transcript was strongly accumulated in flower buds, flowers, and young siliques, but was barely detectable in seedlings, rosette and cauline leaves, and stems (Supplemental Figure S1A). Our results are very similar to the publicly available expression profile of the *GPR1* gene based on RNA-sequencing (RNA-seq) analysis (Supplemental Figure S1B). To investigate the expression of *GPR1* in detail, we generated *ProGPR1:GUS* transgenic lines expressing *GUS* reporter gene under control of the *GPR1* promoter. Consistent with the above results, GUS staining was only detected in flowers at developmental stages 9 to 13 and one day post anthesis, and the GUS signal was restricted to anthers and ovules (Figure 1A,B; Supplemental Figure S2). In anthers, GUS signal was specifically detected in tapetum and developing pollen (Figure 1C,D). In vitro and in vivo pollen germination analysis showed that *ProGPR1:GUS* was also expressed in pollen tubes at high levels (Figure 1E,F). In ovules, *ProGPR1:GUS* was expressed specifically in the embryo sac through stage FG3 to the mature stage of FG7, and its expression was still detectable one day post anthesis (Figure 2).

### 2.2. GPR1 Protein Localizes to Nucleus and Cytoplasm

**Figure 3:**
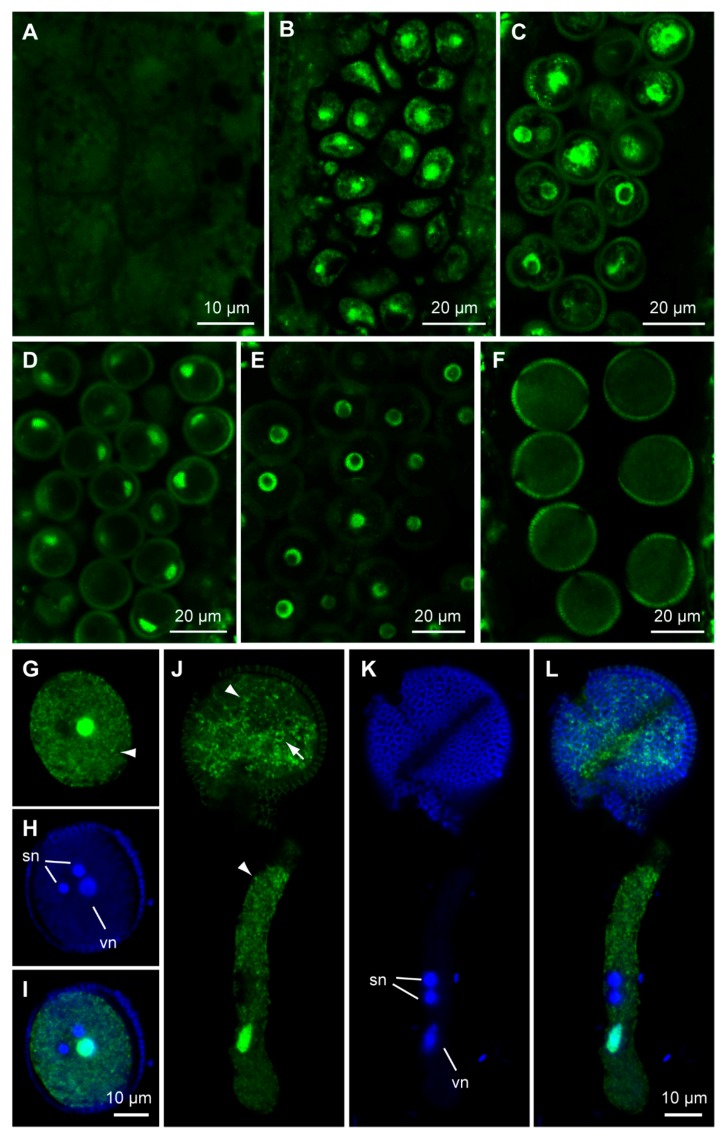
Expression patterns of *ProGPR1:GPR1-EGFP* and *ProLAT52:GPR1-EGFP* during microsporogenesis. (**A**–**F**) *ProGPR1:GPR1-EGFP* expression during microsporogenesis. There is no detectable GPR1-EGFP fluorescence in pollen mother cells (**A**); then strong *GPR1-EGFP* signals were detected in nucleus and cytoplasm of the tetrad (**B**), microspore (**C**), polarized microspore (**D**), and bicellular pollen (**E**); GPR1-EGFP signal was undetectable in mature pollen (**F**). (**G**–**L**) Co-staining of *ProLAT52:GPR1-EGFP* and DAPI in mature pollen (**G**–**I**) and pollen tube (**J**–**L**). (**G**,**J**) *GPR1-EGFP* signals; (**H**,**K**) DAPI stained nuclei; (**I**,**L**) merged images of (**G**,**H**) and (**J**,**K**). sn: sperm nucleus; vn: vegetative nucleus. Figure (**G**) to (**I**) shares the same scale bar as shown in (**I**); Figure (**J**) to (**L**) shares the same scale bar as shown in (**L**). Note GFP signal overlapped with DAPI only in vegetative nucleus.

**Figure 4:**
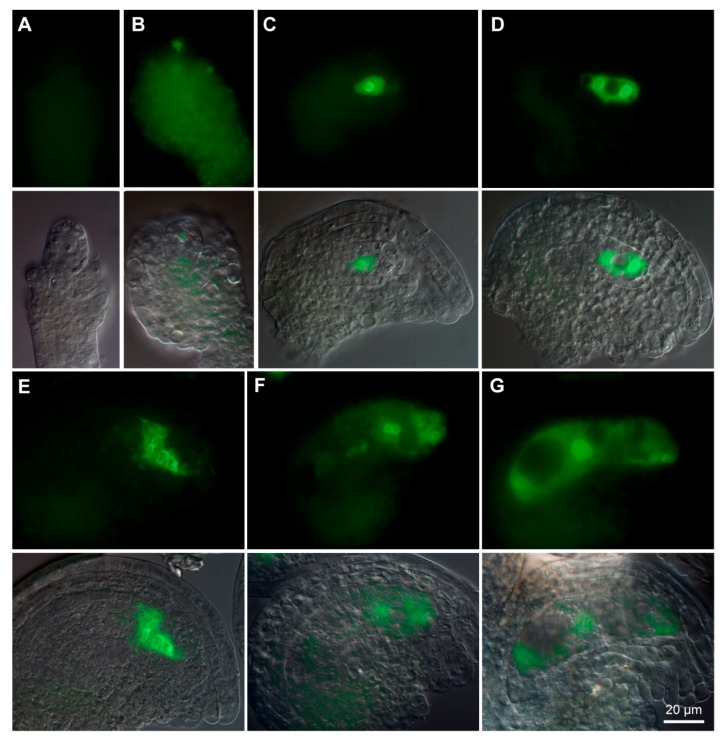
Expression patterns of *ProGPR1:GPR1-EGFP* during female gametophyte development. (**A**) Ovule at stage 2-III. No detectable GPR1-EGFP fluorescence at this stage. (**B**) Ovule at stage 3-I, showing GPR1-EGFP fluorescence in megaspore mother cell. (**C**–**G**) **F**luorescence was detected in nucleus and cytoplasm of FG1 stage embryo sac (**C**); FG3 stage embryo sac (**D**); FG4 stage embryo sac (**E**); FG6 embryo sac (**F**); and FG7 embryo sac (**G**). Figure (**A**) to (**G**) shares the same scale bar as shown in (**G**).

To explore the subcellular localization of GPR1 protein, we generated transgenic Arabidopsis plants expressing either a C-terminal fusion of *GPR1* and *ENHANCED GREEN FLUORESCENT PROTEIN* (*EGFP*) under control of the *GPR1* promoter (*ProGPR1:GPR1-EGFP*), or a N-terminal fusion of *GPR1* and *EGFP* under control of the *35S* promoter (*Pro35S:EGFP-GPR1*). Consistent with the *ProGPR1: GUS* expression patterns, *ProGPR1:GPR1-EGFP* was only expressed in tapetum, developing pollen and ovule (Supplemental Figure S3; Figure 3 and Figure 4). During pollen development, GPR1-EGFP signal was undetectable in pollen mother cell (Figure 3A), then was detected in nucleus and cytoplasm of tetrad, microspore, polarized microspore, and bicellular pollen (Figure 3B–E; Supplemental Figure S3). Furthermore, in bicellular pollen, GPR1-EGFP signal was strong in the vegetative cell nucleus but was undetectable in the germ cell nuclei (Figure 3E; Supplemental Figure S3). In mature pollen, the GPR1-EGFP signal was decreased to below detectable level (Figure 3F). In in vitro germinated pollen tubes, GPR1-EGFP signal was also undetectable (Data not shown). *ProGPR1: GUS* showed strong expression in mature pollen and pollen tube. Therefore, to determine the localization of GPR1-EGFP in mature pollen and pollen tube, we generated transgenic lines expressing *ProLAT52:GPR1-EGFP* (*ProLAT52* is a strong pollen-specific promoter). In mature pollen, ProLAT52:GPR1-EGFP signal was detected in nucleus and cytoplasm of the vegetative cell, but was undetectable in the germ cell nuclei (Figure 3G–I). This is consistent with the expression pattern of *ProGPR1:GPR1-EGFP* in bicellular pollen (Supplemental Figure S3B). In pollen tube, GPR1-EGFP was also only detected in the vegetative nucleus and cytoplasm (Figure 3J–L). Furthermore, the GPR1-EGFP localized to some punctate and ring-like structures in the cytosol of the vegetative cell (Figure 3G,J). During ovule development, GPR1-EGFP signal was undetectable at developmental stage 2-III (Figure 4A), then was first detected in megaspore mother cell at stage 3-I (Figure 4B), and maintains in functional megaspore, coenocytic female gametophyte and mature embryo sac, and also resided in nucleus and cytoplasm (Figure 4C–G). GPR1-EGFP labeled punctate structures was also observed in cytoplasm of female gametophyte (Supplemental Figure S4). We also examined the EGFP-GPR1 signals in root tips of the *Pro35S: EGFP-GPR1* transgenic plants. We found that EGFP-GPR1 also localized to both nucleus and cytoplasm of root cells, and also resided in punctate and ring-like structures (Supplemental Figure S5). The identity of these structures remains to be elucidated.

### 2.3. GPR1 Is Not the Ortholog of the Tobacco NtS2

GPR1 was reported to be the orthologous of the tobacco sperm-cell-expressed protein NtS2 [21]. However, unlike NtS2, GPR1 was not detectable in sperm cell nuclei (Figure 3E,G–L; Supplemental Figure S3B). Therefore, we did amino acid sequence alignment of NtS2 and GPR1. To our surprise, these two proteins only share 20% sequence similarity (Supplemental Figure S6A). We then searched the Arabidopsis database using NtS2 amino acid sequence, and found that it shares 64% similarity with the putative protein At3g23870 (Supplemental Figure S6B).

### 2.4. Isolation and Phenotypic Characterization of gpr1 Mutant

**Figure 5:**
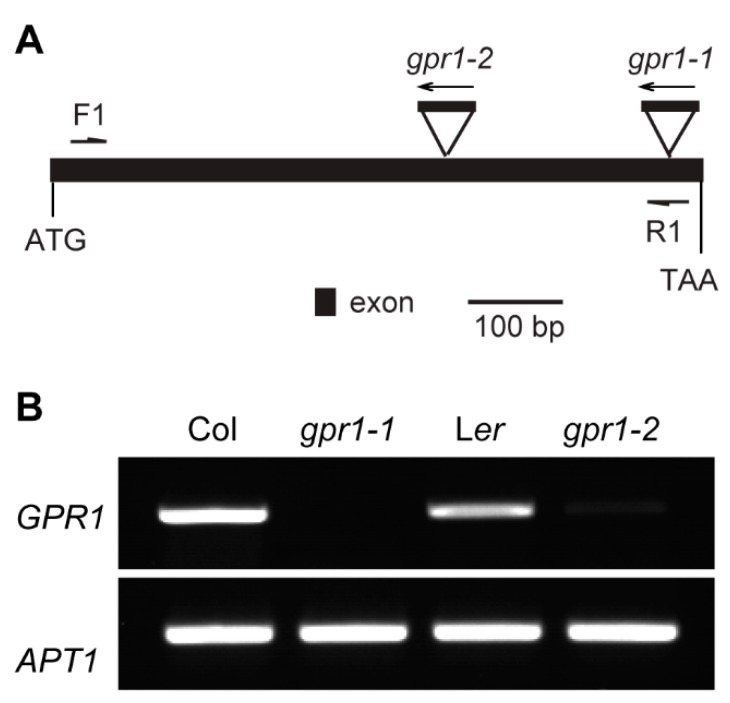
Isolation of *gpr1* mutant. (**A**) Schematic diagram of T-DNA or *Ds* insertion sites in *gpr1* mutants. Dark box indicates exon; triangles indicate T-DNA or *Ds* insertion sites. (**B**) Reverse transcription (RT)-PCR analysis of the expression levels of *GPR1* in wild-type, *gpr1-1*, and *gpr1-2* flower buds. *APT1* was used as the internal control.

**Figure 6:**
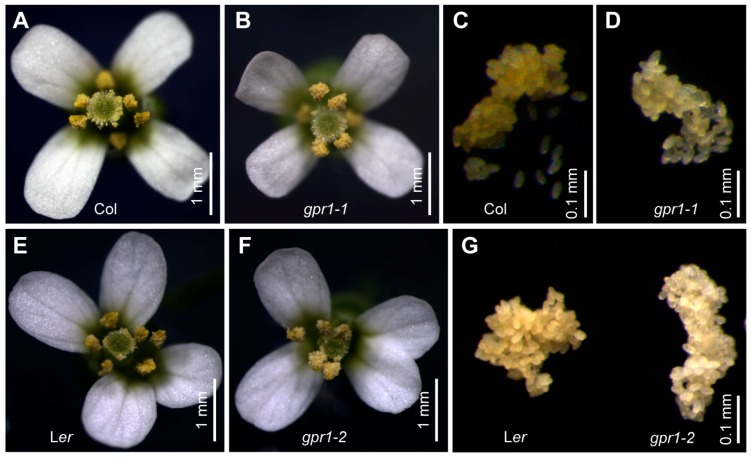
Pollen grains of *gpr1* mutants are pale in color. (**A**,**B**) Flower from Col wild type and *gpr1-1* mutant. (**C**,**D**) Pollen grains collected from Col and *gpr1-1* mutant. (**E**,**F**) Flower from L*er* wild type and *gpr1-2* mutant. (**G**) Pollen grains collected from L*er* and *gpr1-2* mutant. Note pollen grains of *gpr1-1* and *gpr1-2* are pale compared with those of the wild type.

**Figure 7:**
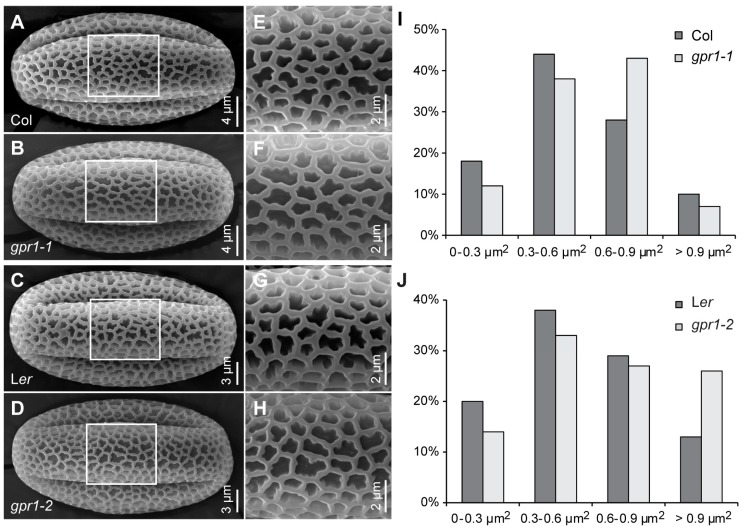
Pollen grain surface of wild-type and *gpr1* mutant. (**A**–**H**) Scanning electron micrographs of the surface of pollen grains from wild type and *gpr1* mutant. (**E**–**H**) Higher-magnification images of rectangular regions in (**A**–**D**), respectively. (**I**,**J**) Lacunae size of pollen grain exine (*n* = 183–193).

To understand the function of Arabidopsis *GPR1*, we obtained T-DNA insertion mutant *gpr1-1* (SALKseq_034266) and *Ds* transposon insertion mutant *gpr1-2* (CSHL_GT24095) (Figure 5A). RT-PCR analyses revealed that no *GPR1* transcripts were detectable in the inflorescence of homozygous *gpr1-1* and *gpr1-2* mutants (Figure 5B). There was no observable difference in vegetative growth between *gpr1* mutants and wild-type plants (Supplemental Figure S7). Although *GPR1* was strongly expressed during pollen and ovule development (Figure 1, Figure 2, Figure 3 and Figure 4), *gpr1* mutant showed no defect in pollen viability, pollen development, or seed set under standard growth conditions (Supplemental Figures S8 and S9), except for its pollen grains exhibiting a pale yellow color compared with wild type (Figure 6). To elucidate why *gpr1* pollen grains exhibited pale color, we examined pollen surface by scanning electron microscopy (SEM) and confocal laser scanning microscope (CLSM). *gpr1* pollen grains displayed a normal patterned but thinner exine surface with thinner muri and larger lacunae compared with wild type (Figure 7; Supplemental Figure S10). These results indicate that the gametophyte-specific *GPR1* is not required for pollen viability and seed set, but may play roles in post gametogenesis process such as pollen coat formation.

### 2.5. GPR1 Loss-of-Function Promotes Pollen Germination and Pollen Tube Growth

To determine whether *GPR1* mutation affects post gametogenesis process, we examined pollen germination in in vitro and in vivo environments. When cultured on pollen germination medium for 8 hours, *gpr1* pollen germinated as efficiently as wild-type pollen (Supplemental Figure S11A–H). However, *gpr1* pollen tubes were significantly longer than that of wild-type (Supplemental Figure S11F,H). To analyze the growth of pollen tubes in vivo, we pollinated wild-type pistils with pollen grains from the *gpr1* mutants or wild-type plant. At 6 h after pollination (HAP), wild-type (Col and L*er*) pollen tubes had just reached the top of the transmitting tract (Supplemental Figure S11I,M). By contrast, *gpr1* pollen tubes had penetrated into the upper part of the transmitting tract (Supplemental Figure S11J,N). At 8 HAP, majority of wild-type pollen tubes were still in the upper part of the transmitting tract, only a few pollen tubes turned toward ovules (Supplemental Figure S11K,O). By contrast, *gpr1* pollen tubes reached more than half of the transmitting tract and most pollen tubes grew to entering the ovules (Supplemental Figure S11L,P).

**Figure 8:**
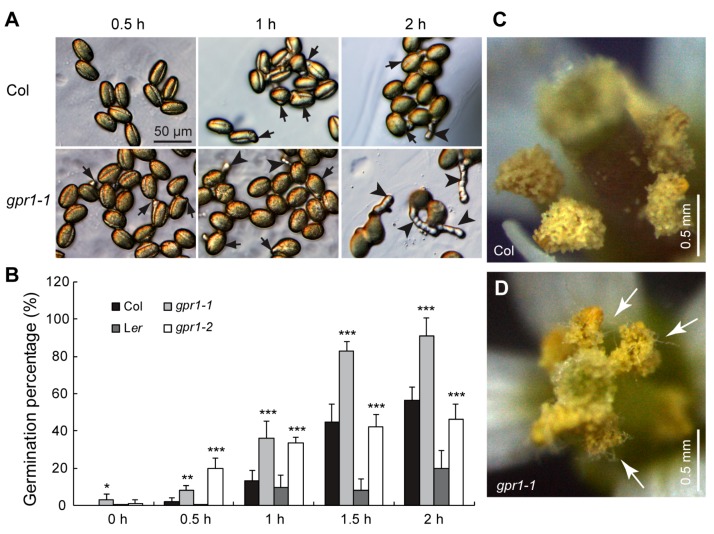
Precocious germination of *gpr1* pollen grains. (**A**) Representative images taken at the indicated time point. Black arrows indicate pollen grains with tube protrusion; black arrowheads indicate pollen grains with tubes equal to or longer than their diameter. (**B**) Pollen germination rate. The data were presented as the means ± SD (*n* = 519–2108; * *p* < 0.05, ** *p* < 0.01, *** *p* < 0.001 by Student’s *t* test). (**C**,**D**) Images of opening flowers of wild type (**C**) and *gpr1-1* (**D**) taken in the afternoon. White arrows indicate pollen tubes. Note precocious pollen germination inside the *gpr1*-1 anther.

**Figure 9:**
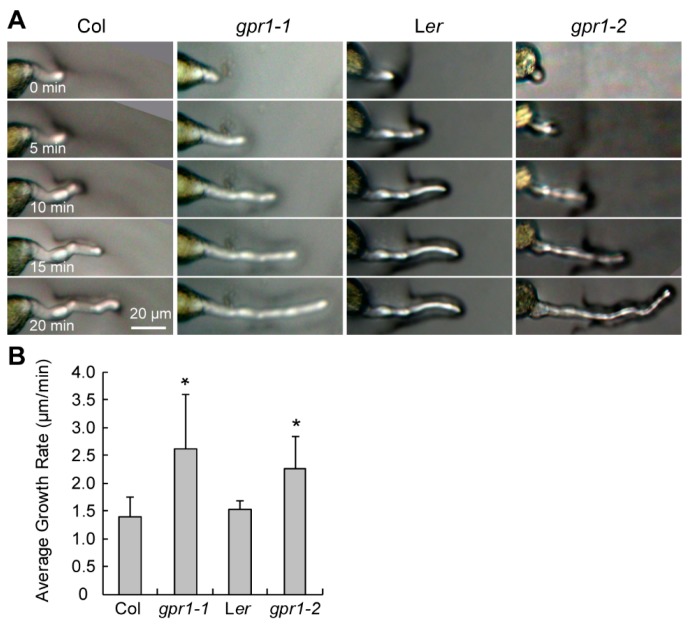
Time-course in vitro pollen germination assay. (**A**) Representative microscope images taken at 5-min intervals. (**B**) Mean growth velocity of WT (Col or L*er*) and *gpr1* mutant pollen tubes. The data were presented as the means ± SD (*n* = 34–53; * *p* < 0.05 by Student’s *t* test).

Longer pollen tubes suggesting hyperactive germination or faster growth velocity. To determine the growth dynamics of *gpr1* pollen tubes, we germinated pollen grains of wild type and *gpr1* plants in vitro for a series of time from 0 to 2 h. Pollen grains of the *gpr1* mutants exhibited hyperactive germination (Figure 8A,B). For example, at 0 h, approximately 3% of *gpr1-1* and 1% of *gpr1-2* pollen grains already exhibited pollen tube protrusion, which was undetectable in wild-type pollen grains (*n* = 1233, 1239, 1286, and 1946 for Col, *gpr1-1*, L*er*, and *gpr1-2*, respectively; Figure 8B). At 0.5 h, approximately 8% of *gpr1-1* and 20% of *gpr1-2* pollen grains had germinated (*n* = 1036 for *gpr1-1*, and 2004 for *gpr1-2*; Figure 8B). By contrast, only approximately 2% of Col pollen grains and 1% of L*er* pollen grains germinated (*n* = 519 for Col, and 2108 for L*er*; Figure 8B). Although precocious pollen germination was not observed inside the dehiscing *gpr1* anthers in the morning (Data not shown), in the afternoon, long pollen tubes can be observed inside dehiscing *gpr1* anthers (Figure 8D). This phenomenon was not observed in the wild-type anther (Figure 8C). We further recorded the in vitro pollen tube elongation by intervals of 5 min, and quantified its growth rate. The results showed that pollen tubes of the *gpr1* mutants elongated faster than those of the wild type (Figure 9).

Taken together, these results indicate that GPR1 functions as a negative regulator of pollen germination and pollen tube growth.

### 2.6. Senescence Marker Expressed Earlier in Unfertilized gpr1 Pistil

**Figure 10:**
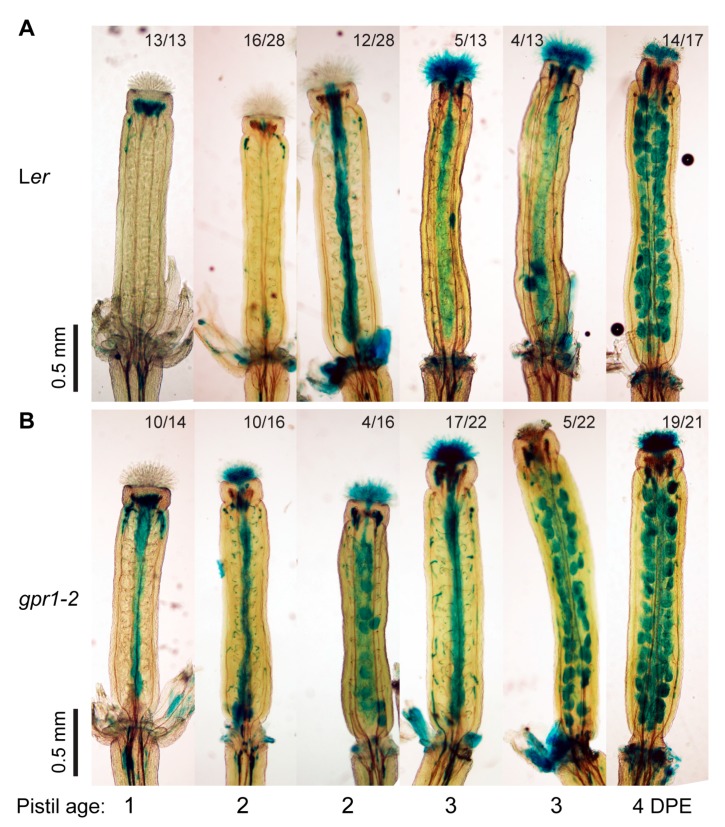
Expression of the senescence marker *P_BFN1_:GUS* in unfertilized wild-type (L*er*) and *gpr1-2* pistil. (**A**) GUS staining in unfertilized pistils of the *P_BFN1_:GUS* line crossed into L*er* background at 0 to 5 day post emasculation (DPE). (**B**) GUS staining in unfertilized pistils of the *P_BFN1_:GUS* line in *gpr1-2*. The number of pistils showing the corresponding GUS expression pattern and total number of pistils examined is indicated above each image. The experiments were repeated three times, shown are the results of one experiment.

When observing the expressions of *ProGPR1:GUS* and *ProGPR1:GPR1-EGFP*, we noticed strong GUS and GPR1-EGFP signals in aborted ovules (Supplemental Figure S12). We then examined the expression of *ProGPR1:GUS* in unfertilized pistils after emasculation. GUS activity was detected specifically in the embryo sac at 0 day post emasculation (DPE); then GUS signal was observed in the whole ovule at 1 to 3 DPE; at 4 DPE, GUS signal was absent in ovules located in the base of the pistil; at 5 DPE some apical ovules also lost the GUS signal (Supplemental Figure S13). This expression pattern of *ProGPR1:GUS* in unfertilized pistil is opposite to that of senescence markers like *SAG12:GUS* and *P_BFN1_:GUS* [19,20,22]. To determine whether *GPR1* mutation affect ovule senescence, we crossed *P_BFN1_:GUS* (in Col background) into both of the *gpr1* mutants and the L*er* wild-type plant (to eliminate background influence). Then we examined *P_BFN1_:GUS* expression in unfertilized pistils of wild-type and *gpr1* mutants. In unfertilized L*er* wild-type pistils, *P_BFN1_:GUS* expression was first observed in the transmitting tract of the 2 DPE pistils; then it was detected in the ovules located in the base of the pistil at 3 DPE; finally, GUS staining was detected in all ovules of pistil at 4 DPE (Figure 10A). Whereas pistil of the *gpr1-2* mutant exhibited GUS staining one-day earlier than pistil of the L*er* wild type (Figure 10B). Similar trend was observed for *gpr1-1* mutant (Data not shown).

### 2.7. GPR1 Mutation Shortens Gametophyte Lifespan

**Figure 11:**
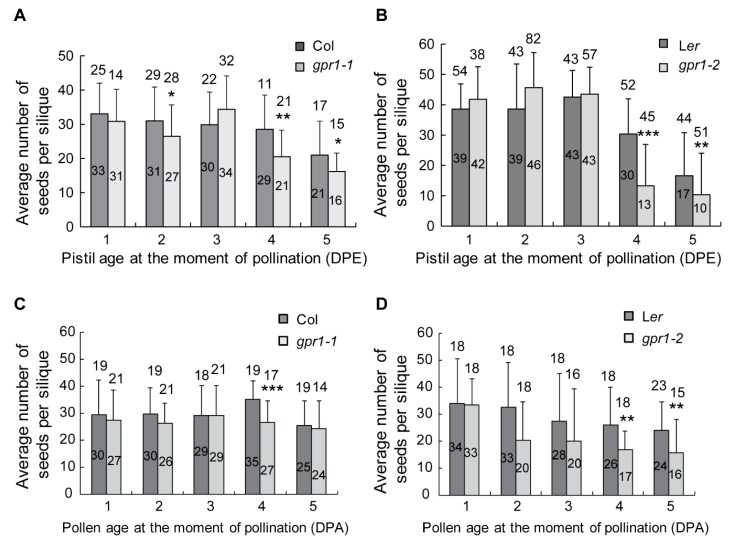
Ovule and pollen lifespan assay. (**A**,**B**) Average seed set of emasculated *gpr1* or wild-type pistils 10 days after hand pollination with wild-type pollen grains. (**C**,**D**) Average seed set of emasculated wild-type pistils 10 days after hand pollination with *gpr1* or wild-type pollen grains. Numbers above the bar indicate total siliques examined; numbers inside the bar represent the average number of seeds per silique. * *p* < 0.05, ** *p* < 0.01, *** *p* < 0.001 by Student’s *t* test.

The above findings suggest that GPR1 may have a role in ovule senescence. To test this, we compared the seed set ability between *gpr1* and wild type after emasculation for 1 to 5 days. When pollinated at 1 to 3 DPE, wild type and *gpr1* pistils produced comparable number of seeds (Figure 11A,B). However, *gpr1* pistils produced significantly less seeds than wild-type pistils when pollinated at 4 to 5 DPE (Figure 11A,B). These results indicate that *gpr1* ovule has a shorter lifespan than that of wild-type plant.

To examine whether *GPR1* mutation also affect pollen longevity, we compared the fertilization efficiency of *gpr1* pollen grain with that of wild type after storage at 4 °C for a series of time of 1 to 5 days. The results showed that pollinations using *gpr1* pollen grains produced less seeds than the ones using wild-type pollen grains (Figure 11C,D). After stored for 4 to 5 days, the difference between wild-type and *gpr1* pollen grains were significant (Figure 11C,D). These results suggest that *gpr1* pollen grains are less viable than that of the wild type after long time storage.

## 3. Discussion

Flowering plants have evolved a highly elaborated reproduction system, which includes gametophyte development, pollination, and fertilization. All these processes are precisely regulated. To date, many genes essential for these processes have been identified and characterized. However, genes that fine-tune these processes are largely unknown. In this study, we show that *GPR1* is specifically expressed in ovule, pollen and pollen tube. GPR1 is not essential for gametogenesis and fertilization, but inhibits hyperactive pollen germination and pollen tube growth, and has positive effects on pollen and ovule longevity. Together, these data suggest that *GPR1* is required for optimal pollination and fertilization processes.

### 3.1. GPR1 Affects Pollen Exine Formation

Pollen grains of angiosperms have an outer exine layer that protect pollen and interact with pistil stigma [23,24,25,26]. Main components of pollen exine are fatty acid derivatives and phenolics [27,28,29]. A growing number of genes have been identified that are important for pollen exine formation, such as *MALE STERILITY2* [30], *CYP703A2* and *CYP704B2* [31,32]; *RUPTURED POLLEN GRAIN1* (*RPG1*), which are involved in fatty acid metabolism [33]; At5g62080 and At5g07230, genes encoding type III lipid transfer proteins [34]; *AtbZIP34*, which encodes a bZIP-family transcription factor [35]; *AtSEC31B*, which encodes a secretory COPII protein [36]. Mutations of these genes usually cause severe pollen exine deposition or patterning defects, and lead to male sterility. Unlike these genes, *gpr1* loss-of-function mutant exhibit normal patterned but thinner exine surface (Figure 7; Supplemental Figure S10). This finding indicates that GPR1 do not affect pollen exine deposition or patterning, but controls the thickness of the exine. Consistent with this function, *GPR1* is specifically expressed during the period of exine synthesis, and is expressed in tapetal cells, which secrete exine materials to the developing microspores (Figure 1 and Figure 3; Supplemental Figure S3).

### 3.2. GPR1 Inhibits Pollen Germination and Pollen Tube Growth

In order to achieve successful fertilization, pollen germination and pollen tube growth are under stringent regulation. To date, a lot of genes essential for pollen germination and tube growth have been identified, and almost all of these genes are positive regulators. However, negative regulators are required to precisely regulate pollen germination and pollen tube growth. So far only a few genes have been identified as negative regulators of pollen germination. Mutations of these genes lead to premature pollen germination within the anther [4,5,6,7,8]. In an attempt to screen genes that are highly expressed in mature pollen but not required for pollen germination and pollen tube growth, Ju et al. identified the *JINGUBANG* (*JGB*) gene [7]. Pollen grains of the *jgb* loss-of-function mutant exhibited normal germination under standard growth conditions, but exhibited hyperactive germination in moist environments [7]. In this study, we identified GPR1 as a negative regulator of pollen germination and tube growth. Loss of GPR1 function leads to accelerated pollen germination and faster tube growth (Supplemental Figure S11; Figure 8 and Figure 9). These findings suggest that GPR1 is required to maintain the optimal pollen germination and tube growth speed. Pollen grains speed up or slow down the tube growth speed via modulation of the plasma membrane H^+^ ATPase activity [37]. JGB inhibits pollen germination through regulating pollen jasmonic acid (JA) synthesis [7]. However, how GPR1 inhibits pollen germination and tube growth need further investigation.

### 3.3. GPR1 Inhibits Ovule and Pollen Senescence

Ovule and pollen longevity is an important factor in determining the ability to produce seeds. The life span of ovule and pollen is species specific and adapted to its ecological requirements [20,38]. Ethylene has been reported to regulate the onset of ovule senescence [20]. In this study, we found that expression pattern of *GPR1* in unfertilized pistils is in contrast to senescence markers *SAG12:GUS* and *P_BFN1_:GUS* (Supplemental Figure S13) [19,20], suggesting that *GPR1* may have a role in ovule senescence. Further emasculation and hand pollination assay showed that loss of GPR1 function shortens both ovule and pollen longevity (Figure 11). Therefore, GPR1 exerts positive effects on ovule and pollen life span.

Although the characterization of the *gpr1* mutants provides insights into the role of the ovule- and pollen-specific *GPR1* gene, the molecular mechanisms of how it negatively regulates pollen germination and tube growth, and how it promotes the longevity of both ovules and pollen grains remain unknown. Investigation of these questions will provide further insights into the molecular basis that fine tunes pollination and fertilization.

## 4. Materials and Methods

### 4.1. Plant Materials and Growth Conditions

T-DNA insertion mutant SALKseq_034266 (*gpr1-1*) is in Col-0 background; and transposon insertion mutant CSHL_GT24095 (*gpr1-2*) is in L*er* background. These mutants were obtained from the Nottingham Arabidopsis Stock Centre (NASC) (Leicestershire, England, UK) and the Cold Spring Harbor Laboratory (CSHL) (Cold Spring Harbor, NY, USA) genetrap collection, respectively. T-DNA or transposon insertion was confirmed by PCR-based genotyping. Primers used are listed in Supplemental Table S1. Plants were cultured under controlled environments at 22 ± 1 °C, 70% relative humidity with a 16 h light/8 h dark photoperiod.

### 4.2. Plasmid Construction and Generation of Transgenic Plants

To create the *ProGPR1:GUS* construct, 2567 bp sequence upstream of the *GPR1* gene start codon were PCR-amplified. The resulting fragment was inserted upstream of the *GUS* reporter gene in the pGreenII0229-GUS vector via *Xho*I and *Spe*I sites [39]. Then, 419 bp of sequence downstream the *GPR1* stop codon (3′ regulating sequence of the *GPR1* gene) was amplified and cloned into the vector downstream of the *GUS* gene. To generate the *ProGPR1:GPR1-EGFP* translational fusion construct, the *GPR1* promoter and full-length coding sequence (nucleotides −2567 to 690 from ATG) was amplified and inserted into the pGreenII0229-EGFP plasmid in frame with the *EGFP* gene [40]. Then, the 3′ regulating sequence of the *GPR1* gene (419 bp) was cloned into the above constructed vector at downstream of the *EGFP* gene. To generate the *ProLAT52:GPR1-EGFP* construct, the 666 bp promoter fragment of the *LAT52* gene was amplified from *Solanum lycopersicum* and cloned into the pGreenII0229-GPR1-EGFP vector upstream of the *GPR1* coding sequence. To construct the p35S:EGFP-GPR1 plasmid, the *EGFP* fragment was cut from the pGreenII0229-EGFP plasmid using *Xho*I and *Sac*I enzymes, then inserted into the pBA002 vector (with CaMV 35S promoter; from Nam-Hai Chua) using the same sites; then *GPR1* full-length cDNA was cloned into the pBA002-EGFP vector at downstream of the *EGFP* gene and in frame with *EGFP*.

All constructed vectors were introduced into *Agrobacterium tumefaciens* strain C58C1 (pMP90/pJIC Sa-Rep) and transformed into wild-type Arabidopsis (Col-0) plants by floral dip method [41]. Seeds harvested from the transformed plants were sowed in soil and screened by spraying 10 mg L^−1^ Basta (Sangon Biotech, Shanghai, China). Basta resistant plants were verified by PCR. Homozygous lines (T3 generation) were obtained from several independent transformants to perform analyses. All primers used are listed in Supplemental Table S1.

### 4.3. RT-PCR Analysis

RT-PCR was performed to test the transcription levels of the *GPR1* gene in mutant or wild-type plants. Total RNA was extracted from inflorescences of 5-week-old plants using Trizol reagent. The first-strand cDNA was synthesized using EasyScript First-Strand cDNA Synthesis SuperMix (TransGen Biotech, Beijing, China). The *APT1* gene was used as an internal control for equal loading. The primer sequences are listed in Supplemental Table S1.

### 4.4. GUS Histochemical Staining

For GUS staining, various tissues of *ProGPR1:GUS* transgenic plants were incubated for 12 h at 37 °C in GUS staining buffer as previously described [40]. After staining, the tissue was cleared in chloral hydrate:distilled water:glycerol (8:3:1, *w*:*v*:*v*). Inflorescences were examined using a Leica M165FC stereomicroscope. Developing ovules were dissected and observed under an Olympus BX63 microscope using differential interference contrast (DIC) optics. Ovule developmental stages were determined as described previously [42].

### 4.5. Scanning Electron Microscopy and Fluorescence Microscopy

For scanning electron microscopy, flowers from wild-type, *gpr1-1* and *gpr1-2* plants were dissected; pollen grains were subjected to freeze drying, then mounted for coating with gold and observed on a QUANTA 200 (FEI, Hillsboro, OR, USA) scanning electron microscope. For observation expression pattern of GPR1-EGFP in ovules and anthers, flowers were dissected at different developmental stages and mounted in an 8% glycerol solution. Fluorescent signals were detected by confocal laser scanning microscope (CLSM, Leica TCS SP5, Wetzlar, Germany).

### 4.6. Pollen Grain and Pollen Tube Staining

To assay pollen viability, Alexander staining was carried out as described [43], and then cleared in Herr’s solution [44] for 2 h. For DAPI and callose staining, flowers were fixed in ethanol:acetic acid (3:1) solution for 2 h, and treated with 2 N NaOH for 10 min. After washing with 50 mmol·L^−1^ K_2_HPO_4_-KOH buffer (pH 7.5) for three times, pollen grains were immersed with 1 g·L^−1^ DAPI (Sigma-Aldrich, Steinheim, Germany) for staining DNA. The stained nuclei were detected using a Leica DM2500 microscope (Leica, Wetzlar, Germany) with a UV filter.

To visualize the in vivo geminated pollen tubes, aniline blue staining was performed as described previously [45]. Emasculated wild-type flowers were pollinated either with wild type or *gpr1* pollen grains. Six or eight hour after pollination, the pistils were fixed in acetic acid:chloroform:ethanol (1:3:6) for 2 h. Then, the pistils were washed in distilled H_2_O, and softened overnight in 8 N NaOH. After the softening step, the pistils were washed three times with distilled H_2_O, and then stained with aniline blue solution (0.05% aniline blue in 0.1 mol·L^−1^ potassium phosphate buffer, pH 7.5) for 3 h by avoiding light. After staining, pistils were washed with 0.1 mol·L^−1^ potassium phosphate buffer, and mounted on a microscope slide, and gently squashed under a cover slip. The pistils were observed using a Leica DM2500 microscope with a UV filter.

### 4.7. In Vitro Pollen Germination Analysis

In vitro pollen germination was performed according to previously published methods [46]. Briefly, pollen grains collected from newly opened flowers were placed on pollen germination medium [1 mmol·L^−1^ CaCl_2_, 1 mmol·L^−1^ Ca(NO_3_)_2_, 1 mmol·L^−1^ MgSO_4_, 0.01% (*w*/*v*) H_3_BO_3_, and 18% (*w*/*v*) sucrose, solidified with 0.5% (*w*/*v*) agar, pH 7.0]. The plates were cultured at 28 °C under moist conditions. After 8 h incubation, digital images were collected with a Leica DFC420 CCD camera equipped on a Leica M165FC stereomicroscope (Leica, Wetzlar, Germany). Pollen germination percentage and the length of the pollen tubes were measured using ImageJ software. Protrusion from the aperture was regarded as positive germination [47].

### 4.8. Female and Male Gametophytes Longevity Test

To test the longevity of ovules, flowers one day before anthesis from *gpr1* mutant and wild type were emasculated, then the pistils were hand pollinated with freshly harvested wild-type pollen grains at 1 to 5 days post emasculation. Ten days after pollination, siliques were harvested and seeds were counted. For pollen lifespan analysis, mature pollen grains were harvested from opening flowers of *gpr1* mutants and wild type, and were stored at 4 °C for 1 to 5 days before pollinated to pistils of freshly emasculated wild type flowers. Ten days after pollination, siliques were harvested and seeds were counted.

## Supplementary Materials

Supplementary materials can be found at www.mdpi.com/1422-0067/18/6/1303/s1.

## Abbreviations

DPA	Day post anthesis
DPE	Day post emasculation
Hag	Hour after germination
Hap	Hour after pollination
