# Supplementary material for: The Arabidopsis GPR1 Gene Negatively Affects Pollen Germination, Pollen Tube Growth, and Gametophyte Senescence
Source: Int J Mol Sci. 2017 Jun 21;18(6):1303. doi: 10.3390/ijms18061303 (PMC5486124; doi:10.3390/ijms18061303)
Supplement: Supplementary file 1 [file ijms-18-01303-s001.pdf]

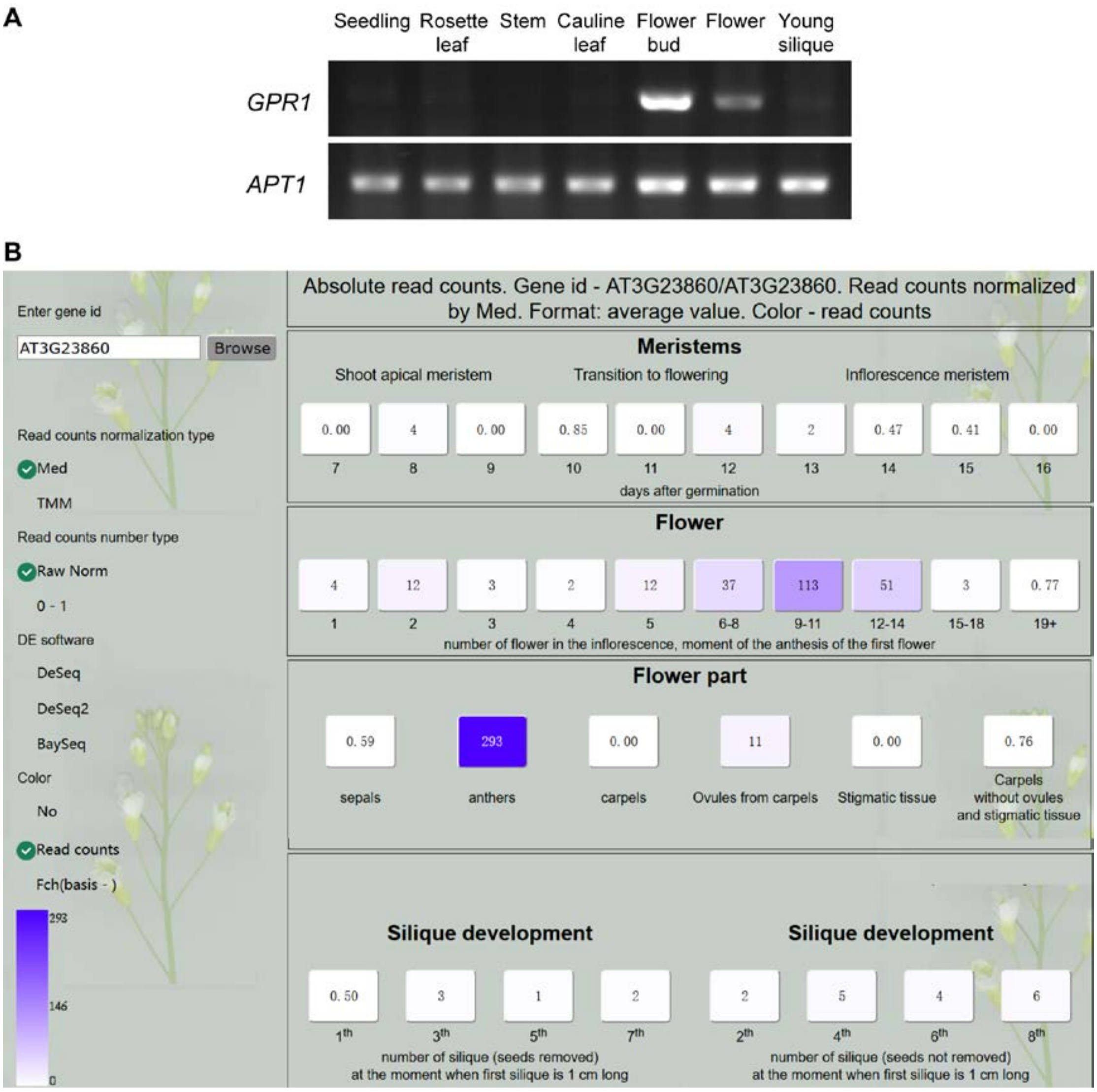

Supplemental Figure S1. Transcription levels of *GPR1* in various Arabidopsis tissues. **(A)** *GPR1* expression analysis by RT-PCR. *APT1* was used as internal control. **(B)** *GPR1* expression profile based on RNA-seq analysis. Data were downloaded from the "Transcriptome Variation Analysis" database (TRAVA, http://travadb.org/).

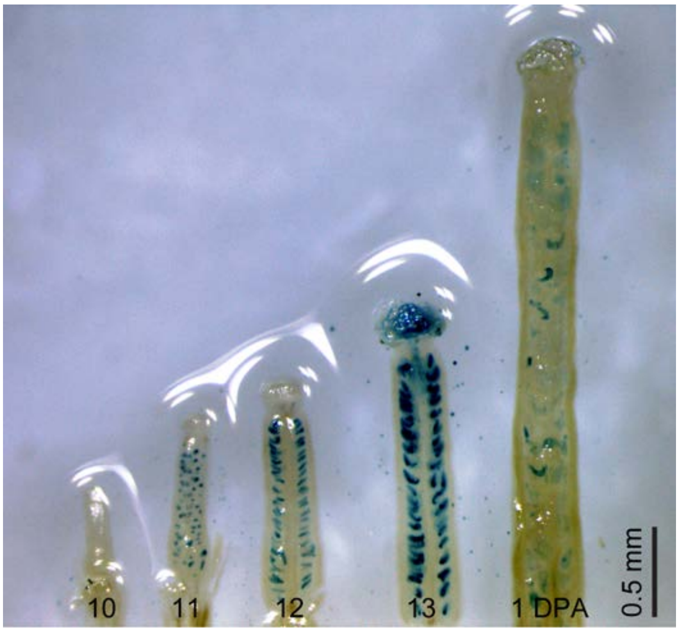

Supplemental Figure S2. Expression of *ProGPR1:GUS* in pistils and young silique at 1 day post anthesis (DPA). GUS staining was undetectable in pistil of stage 10 flower. Increasing GUS signals were detected in ovules of stage 11 to 13 pistils, and then decreased in 1 DPA silique.

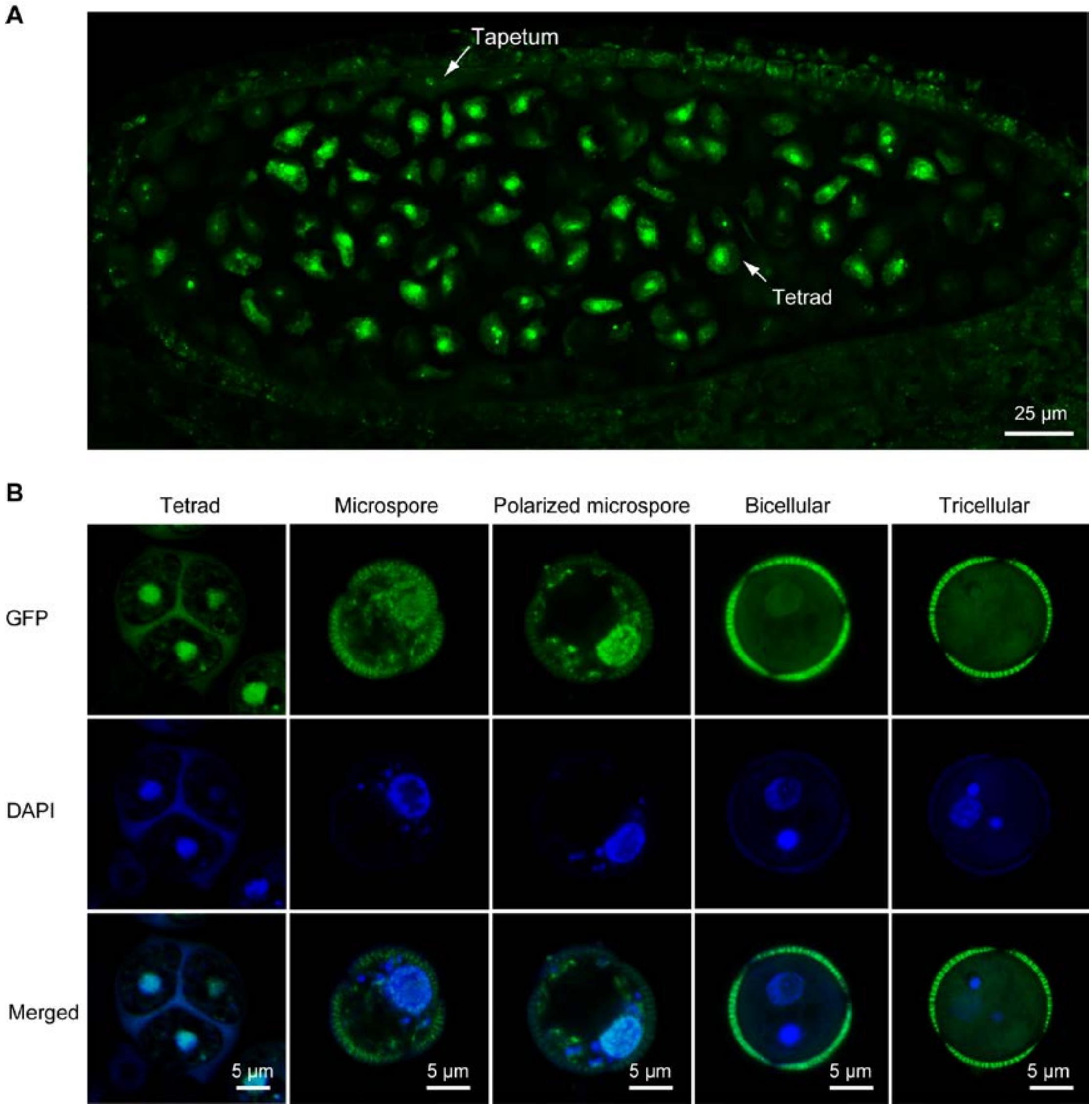

Supplemental Figure S3. *ProGPR1:GPR1-EGFP* expression in anther at stage 7 and during pollen development. **(A)** In stage 7 anther, GPR1-EGFP fluorescence was detected in tapetal cells and tetrads, and the fluorescence was strong in the nucleus. **(B)** *ProGPR1:GPR1-EGFP* expression during pollen development. Nucleus was visualized by DAPI staining. Note GFP signal overlapped with DAPI only in vegetative nucleus of bicellular pollen grain.

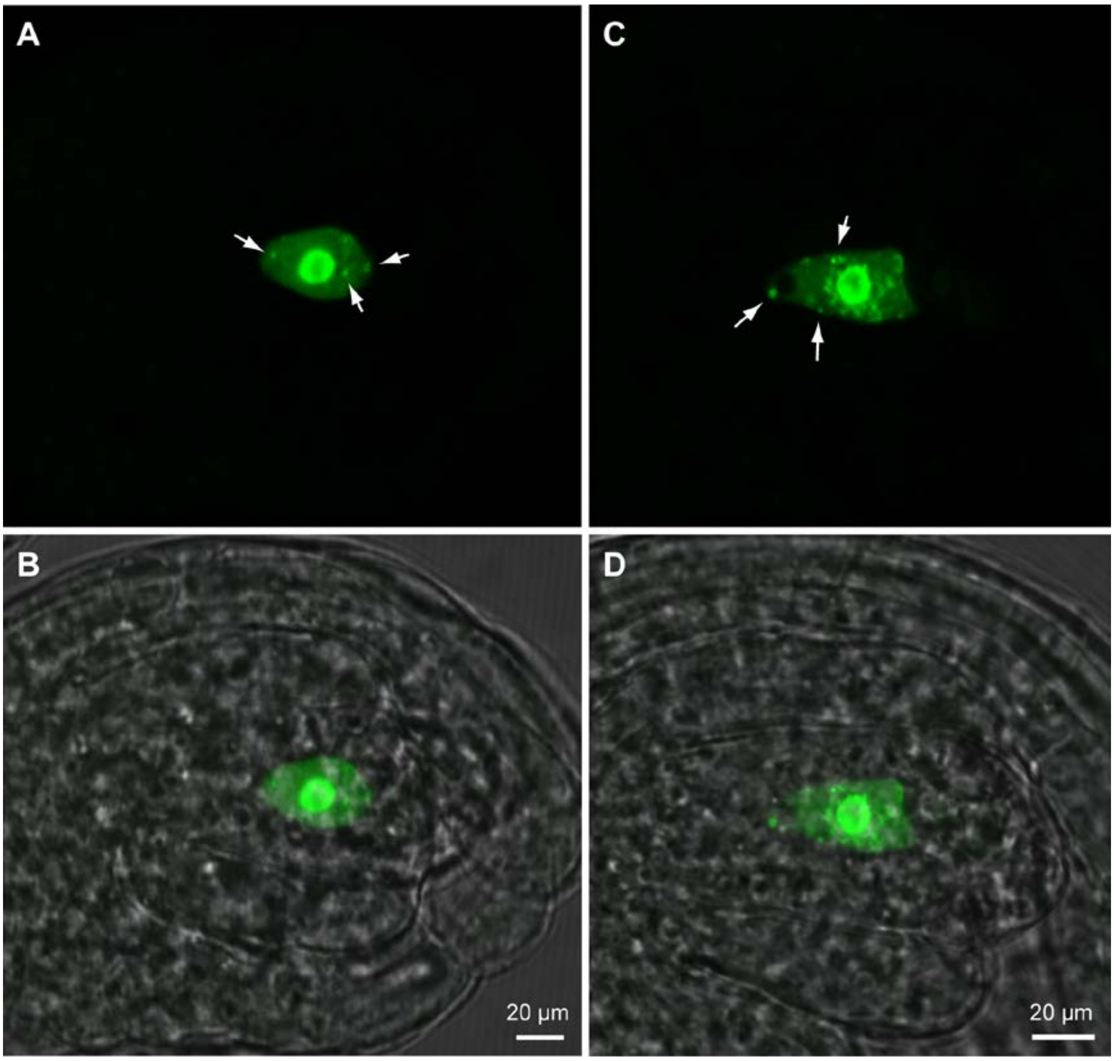

Supplemental Figure S4. Subcellular localization of GPR1-EGFP in female gametophyte. **(A-D)** Ovules at FG1 stage from *ProGPR1:GPR1-EGFP* transgenic plants were observed using confocal laser scanning microscope. Shown are GPR1-EGFP images **(A, C)** and merged images of GFP channel and bright field (B, D). Arrows indicate punctate structures.

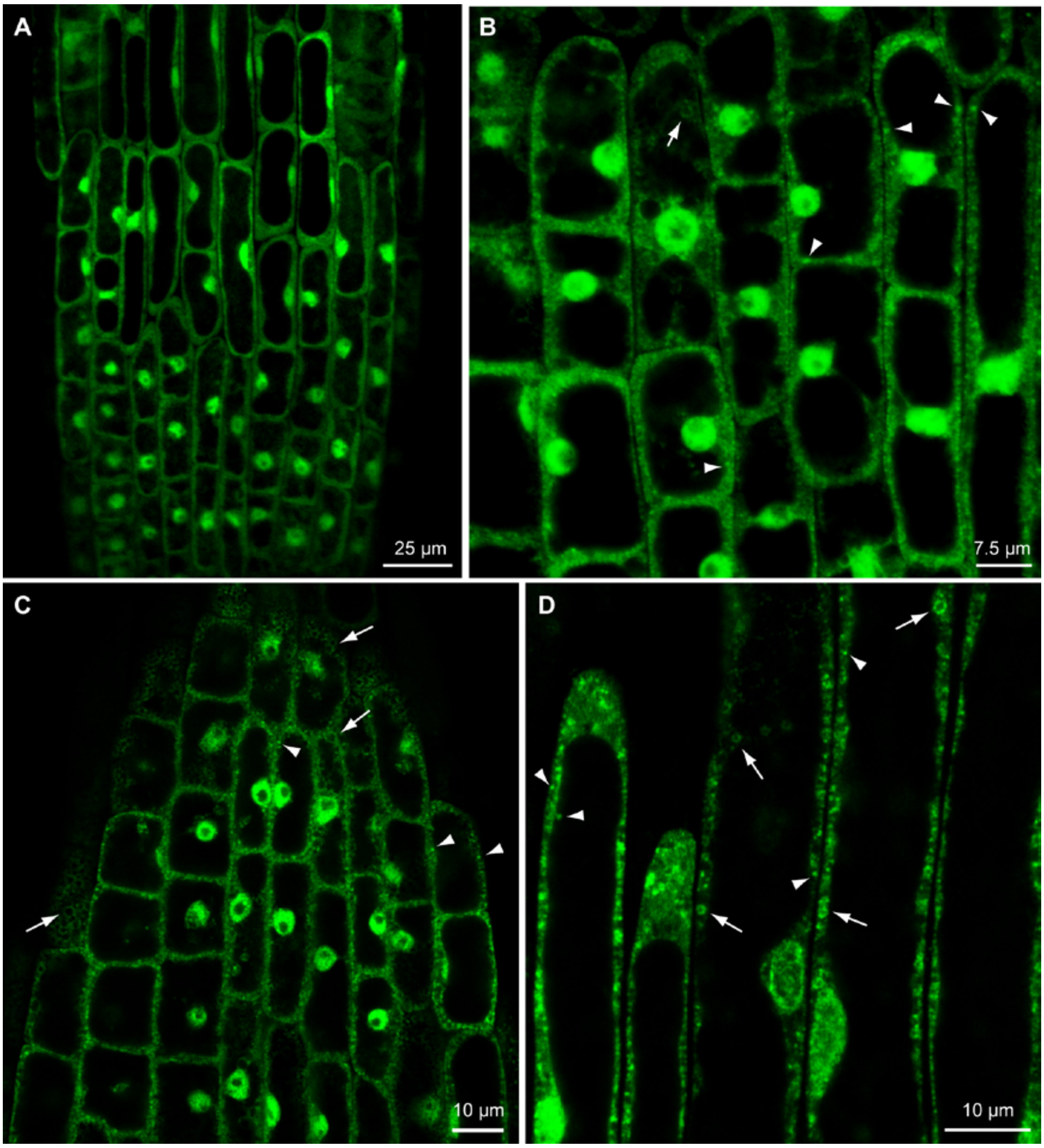

Supplemental Figure S5. Subcellular localization of EGFP-GPR1 in Arabidopsis root tip cells. **(A)** Confocal image of root tip of *Pro35S:EGFP-GPR1* seedling. **(B, C)** Enlarged image of root tip cells in the transition zone. **(D)** Enlarged image of root tip elongated cells. Note fluorescence was detected in cytoplasm and nucleus. Arrowheads indicate punctate structures; arrows indicate ring-like structures.

A

```
NtS2            YIGICSLAGSLTVMGVKAIGIAMKLTFGGQNQFKYFETWFFIIFVLIFCLLQ--------  52
GPR1            ------MEK--NIFKKISAVVPTELDFDRAIRFEYQIPNCTLI-PDRVCHDDISDIRQKY  51
                      :    .::   :  :  :* *.   :*:*      :*    .*  :

NtS2            -----------LNYLNKALDTFNT-AVVSPIYYVM-------FTTL----------TIVA  83
GPR1            AVKVMSAGTTLSNKLNDVLHEFPCVRHLDPVYASLLHQRYNMYHYDRAVRQVSVTQTLVN  111
                            * **..*. *     :.*:*  :       :             *:*

NtS2            SM-IMFKDYVHQNATQIITELCGFVTIL-----------C--------------------  111
GPR1            VMSFNYVDLLRKDDDCDSRDKCRSLGVTALARMLTFAKSCIPALNLLDQVREFMASVEDD  171
                 * : : * ::::      : *  : :            *

NtS2            --GTFLLHKTKDMGSNPSKPIPVLLPKTDTDCKPTSETTKIPAEV--------------  154
GPR1            AAATSLVNKYSDVLPPEKYPFPDIVWEDETADFVDPETLLLVEELDRRYELWLKGGSHI  230
                  .* *::* .*:    . *:* :: : :*      **  :  *:
```

B

```
NtS2            ------------------------------------------------------------ 0
AT3G23870       MDQMSPDNINGVILAVSSSIFIGSSFIIKKKGLKKAGASGVRAGEGGYGYLKEPWWWAGM 60


NtS2            ------------------------------------------------------------ 0
AT3G23870       ITMIVGEVANFAAYAFAPAILVTPLGALSIIFSAVLAHFILKEKLHMFGILGCILCVVGS 120


NtS2            ---------------------------------------------------------YIG 3
AT3G23870       TTIVLHAPHEQKIESVKQIWQLAIEPGFLVYSAVIVIVVAILIFYYEPRYGKTHMIVYVG 180
                                                                         *:*

NtS2            ICSLAGSLTVMGVKAIGIAMKLTFGGQNQFKYFETWFFIIFVLIFCLLQLNYLNKALDTF 63
AT3G23870       ICSLMGSLTVMSVKAVAIAIKLTFSGTNQFKYFNTWIFILVVATCCILQINYLNKALDTF 240
                **** ******.***:.**:****.* ******:**:**:.*   *:**:**********

NtS2            NTAVVSPIYYVMFTTLTIVASMIMFKDYVHQNATQIITELCGFVTILCGTFLLHKTKDMG 123
AT3G23870       NTAVISPVYYVMFTTFTIIASMIMFKDWASQSGLKIATELCGFVTILSGTFLLHKTKDMG 300
                ****:**:*******:**:********:. *.. :* **********.************

NtS2            SNPSKPIPVLLPKTDTDCKPTSETTKIPAEV----  154
AT3G23870       NSASGRGSISMPTRDTPVFTNSGSGRSSSSDKVAS  335
                .. *    : :*. **    .* : :  :.
```

Supplemental Figure S6. Amino acid sequence alignment of NtS2, GPR1 and At3g23870 proteins. **(A)** Amino acid sequence alignment of NtS2 and GPR1. **(B)** Amino acid sequence alignment of NtS2 and At3g23870. The alignment was performed online at http://www.ebi.ac.uk/Tools/msa/clustalo/.

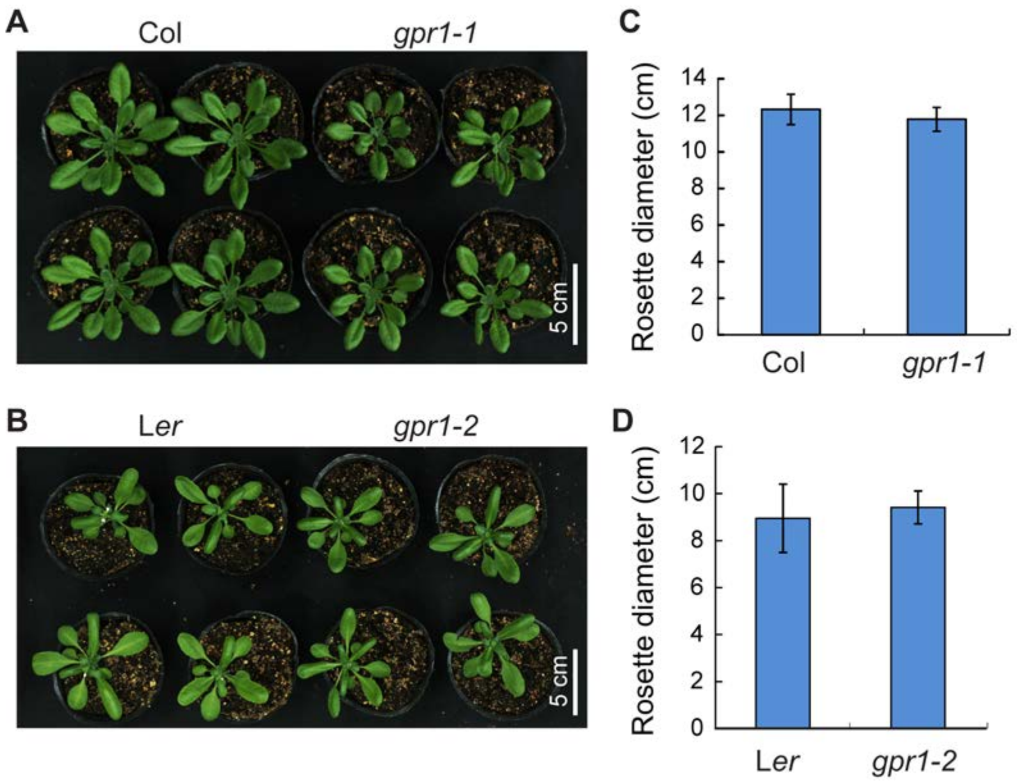

Supplemental Figure S7. Phenotypes of *gpr1* mutants. **(A, B)** Rosette of 4-week-old plants. **(C, D)** Rosette diameter of 5-week-old plants. The data are presented as means ± SD (n = 8, no significant differences between *gpr1* mutants and wild type by Student's *t* test).

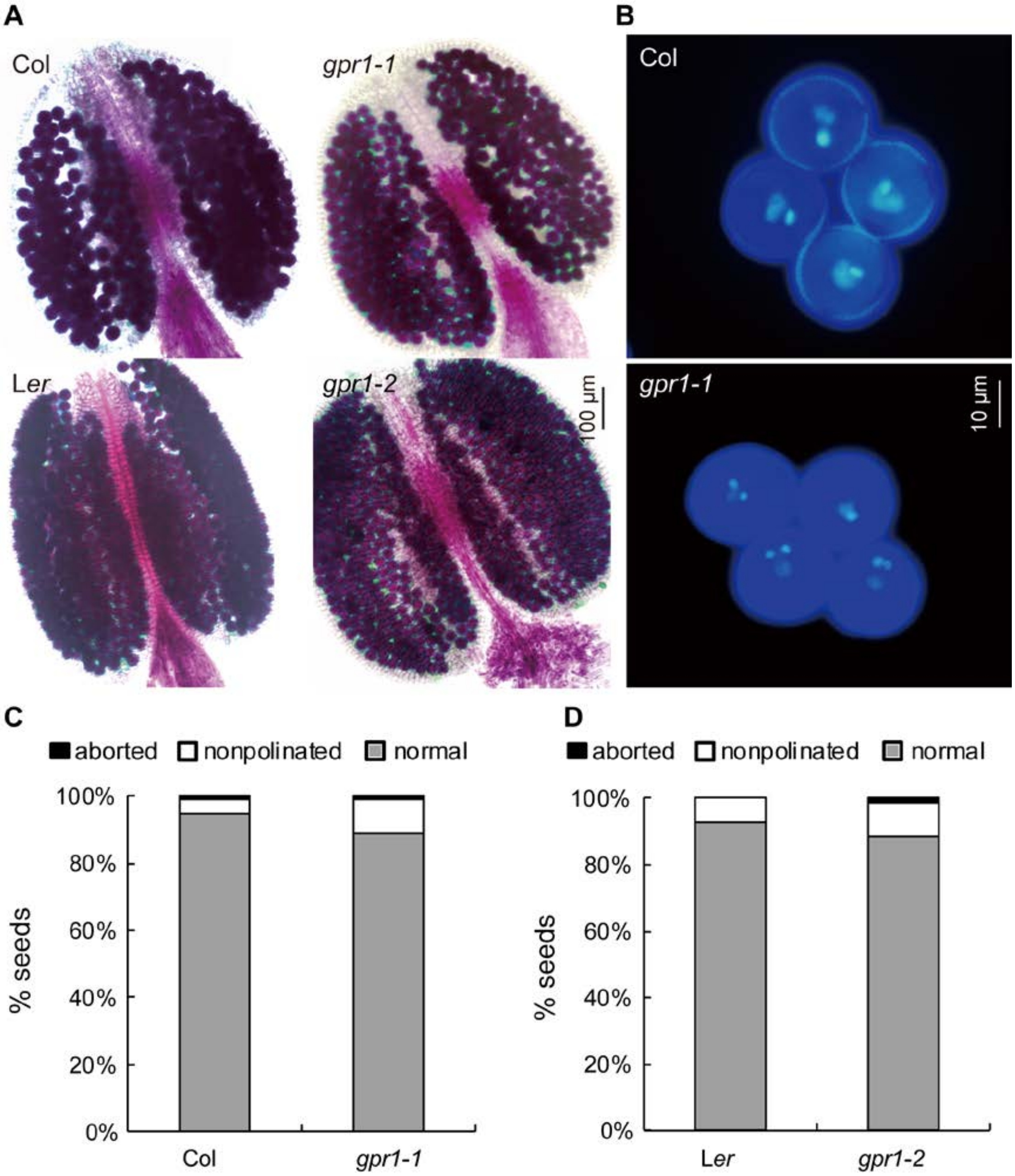

Supplemental Figure S8. Viability analysis of *gpr1* pollen grain. **(A)** Alexander stained anthers from wild-type and *gpr1* plants. Pollen with normal viability was stained in purple. **(B)** DAPI stained pollen grains. **(C, D)** Quantification of seed setting after crossing with *ms1-1*. *ms1-1* pistils were hand-pollinated with pollen grains from wild-type or *gpr1* plants. Siliques were harvested at 10 d after pollination and the seeds were counted. The data are presented as means ± SD (n = 8 siliques for each crossing, no significant differences between *gpr1* mutants and wild type by Student's *t* test).

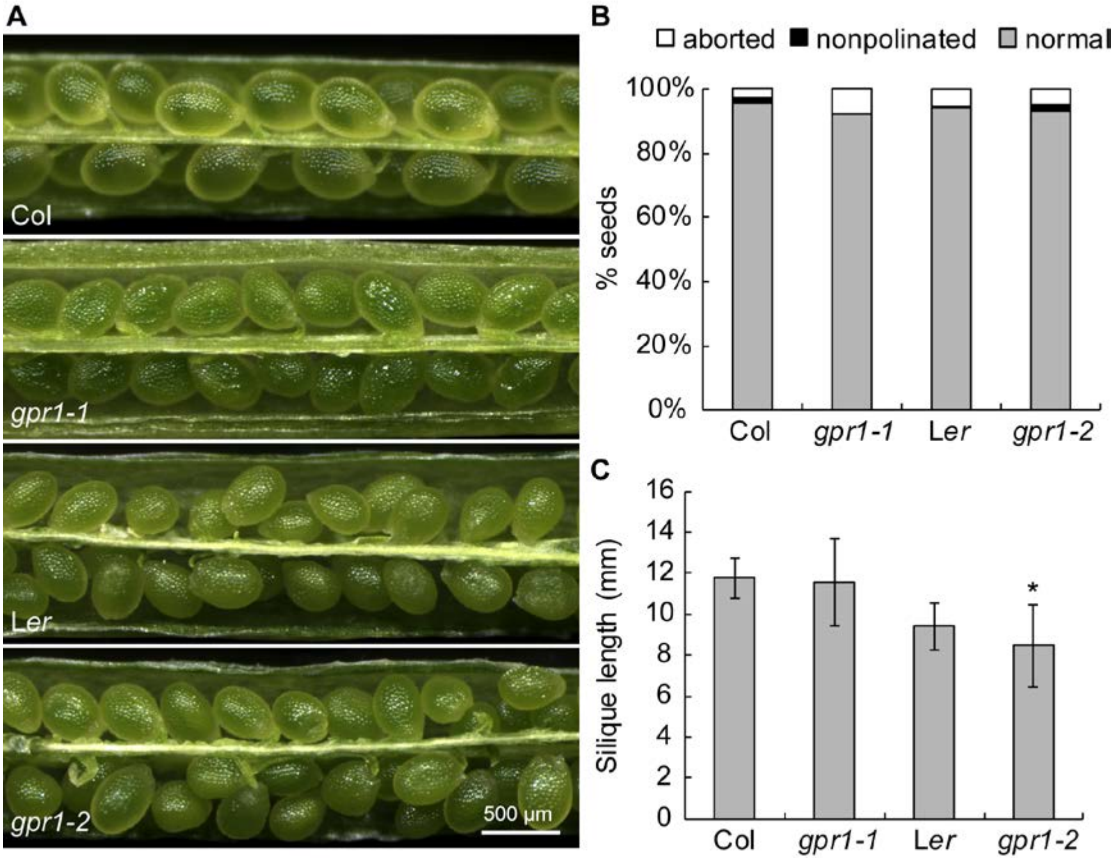

Supplemental Figure S9. Seed setting of *gpr1* mutants. **(A)** Siliques 10 d after pollination from wild-type and *gpr1* mutants. **(B, C)** Quantification of normal and aborted seeds and unpollinated ovules, and silique length. The results are presented as means ± SD (n = 18 siliques for each genotype). Differences between the wild-type and mutants are significant at $P < 0.05$ (*) by Student's *t* test.

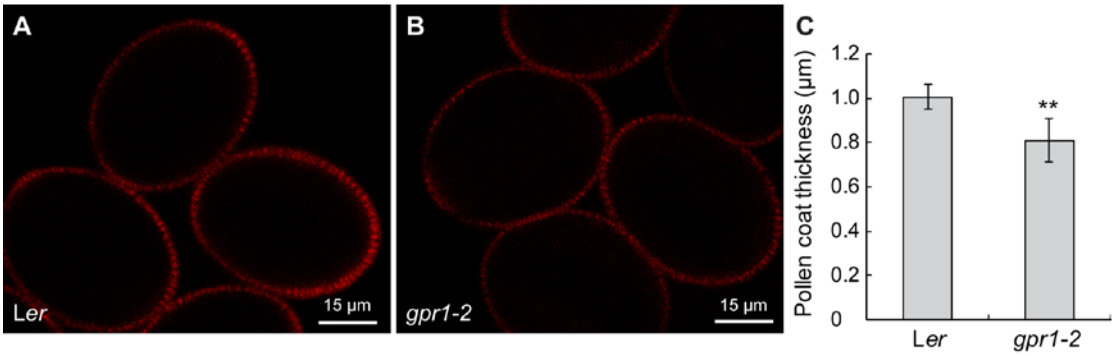

Supplemental Figure S10. Pollen coat analysis of *gpr1-2* mutant. **(A, B)** Fluorescent images taken using a TRITC filter for autofluorescent (red) of pollen coat in L*er* **(A)** and *gpr1-2* **(B)**. **(C)** Pollen coat thickness of L*er* and *gpr1-2*. The data are presented as means ± SD (n = 81 for each genotype, ** P < 0.01 by Student's t test).

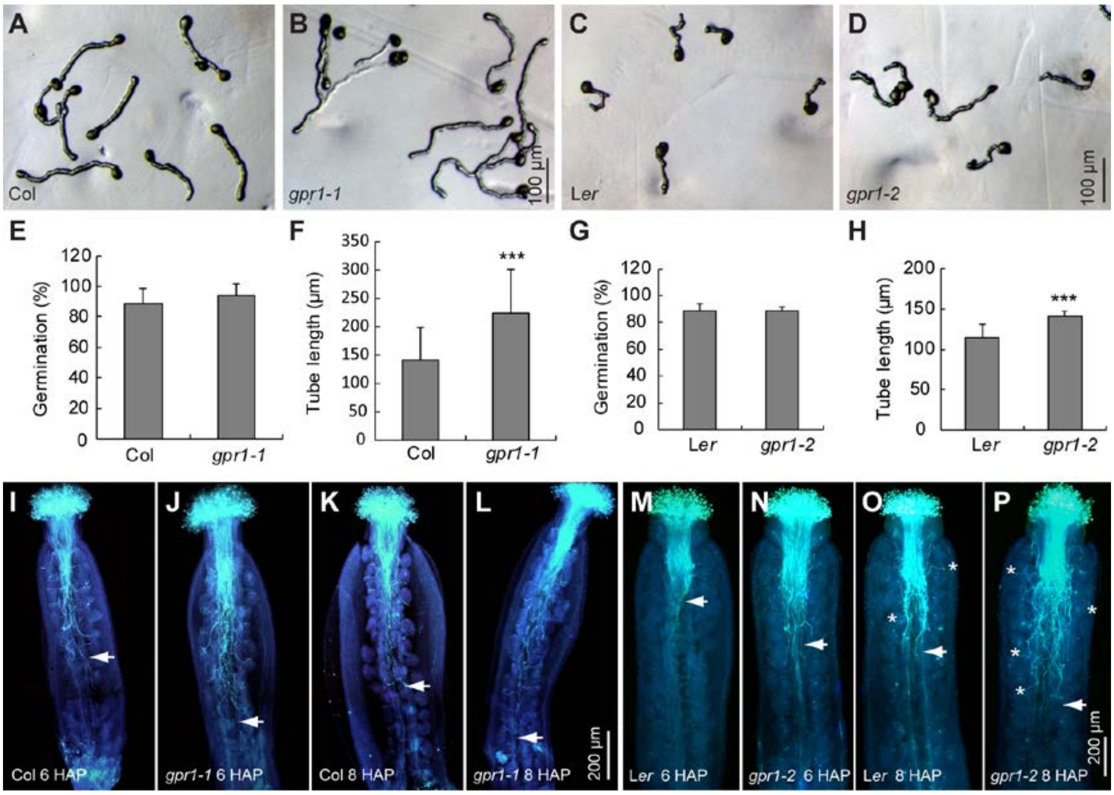

Supplemental Figure S11. In vitro and in vivo pollen germination analysis of *gpr1* mutants. **(A-D)** Pollen grains from Col **(A)**, *gpr1-1* **(B),** L*er* **(C)** and *gpr1-2* **(D)** incubated on germination medium for 8 h. **(E-H)** Pollen germination percentage **(E, G)** and pollen tube length **(F, H)** at 8 h (n = 1390-1674 for Col and *gpr1-1*, n = 170-250 for L*er* and *gpr1-2*; *** $P < 0.001$ by Student's *t* test). **(I-L)** Aniline blue staining of Col pistil 6 h or 8 h after hand pollination with either Col **(I, K)** or *gpr1-1* pollen grains. **(M-P)** Aniline blue staining of L*er* pistil 6 h or 8 h after hand pollination with either L*er* **(M, O)** or *gpr1-2* **(N, P)** pollen grains. Arrows indicate pollen tubes in transmitting tracts. Asterisks indicate pollen tubes that invade the ovule.

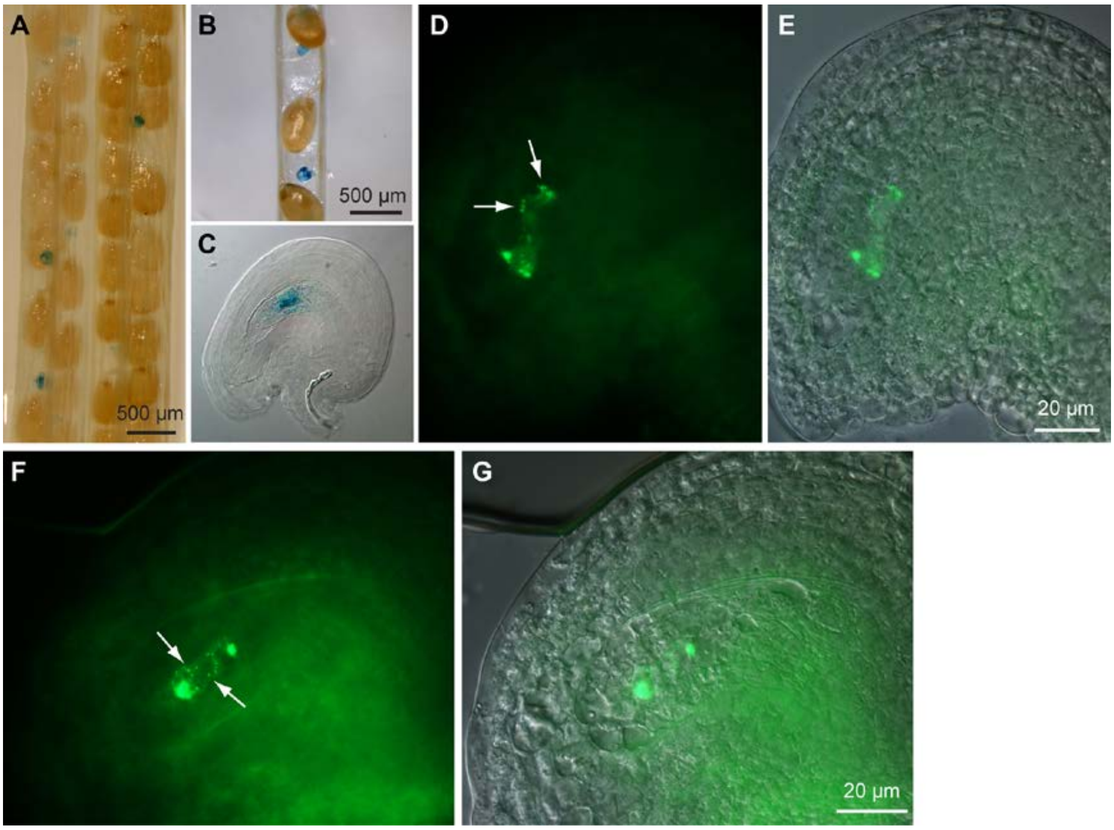

Supplemental Figure S12. Strong *ProGPR1:GUS* or *ProGPR1:GPR1-EGFP* signals were detected in unpollinated or aborted ovules. **(A, B)** *ProGPR1:GUS* siliques, showing GUS signals in unpollinated ovules. **(C)** An aborted ovule from stage 13 flower. Note strong GUS staining in the center of the embryo sac. **(D-G)** Fluorescence **(D, F)** and merged **(E, G)** images of aborted ovules from stage 13 flower of *ProGPR1:GPR1-EGFP* plants. Note strong fluorescent signals in the degenerated embryo sac. Arrows indicate punctate structures.

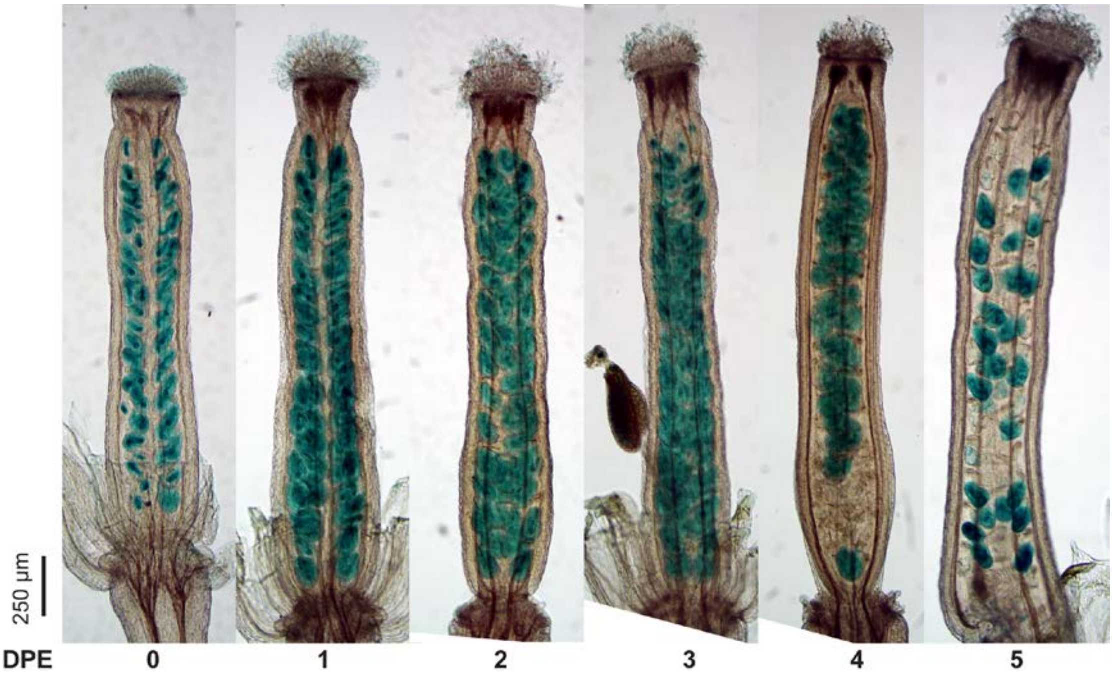

Supplemental Figure S13. Expression of *ProGPR1:GUS* in unfertilized pistils. At 0 to 3 day post emasculation (DPE), GUS activity was detectable in ovules of the whole pistil. At 4 DPE, GUS signal was absent in ovules located in the base of the pistil, and this trend progressed acropetally on the next day.

Supplemental Table S1. Primers used in this study.

Primer Purpose	Primer name	sequence 5'→ 3'
Genotyping	LBa1	TGGTTCACGTAGTGGGCCATC
	Ds5-1	ACGGTCGGGAAACTAGCTCTAC
	GPR1_gF_386	GAAAGGACGATGATTGCGACTC
	GPR1_gR_1402	CTGCCATTGAAGGATAAGAAACTAA
GPR1 promoter cloning	GPR1_F_-2567_XhoI	GCCCTCGAGCAGCCAGAAATATGAAACTATCCCA
	GPR1_R_-1_SpeI	GCATACTAGTTGTTCCACAACGAACCATCTTGAAT
GPR1 terminator cloning	GPR1_F_694_NotI	GTAAGCGGCCGCGGAACCCATTCCAATTTAAACCTCA
	GPR1_R_1112_SacI	GCTGGAGCTCGCCAGAGCAGAGGAACCAATAGTGT
GPR1 genomic DNA cloning	GPR1_F_-2567_XhoI	GCCCTCGAGCAGCCAGAAATATGAAACTATCCCA
	GPR1_R_690_SpeI	CCATACTAGTTATATGGCTTCCACCTTTGAGCCAG
GPR1 cDNA cloning	GPR1_gF_1_EcoRI	ATATGAATTCATGGAGAAGAACATATTCAAGAAGA
	GPR1_gR_693_BamHI	ATATGGATCCTTATATATGGCTTCCACCTTTGAGC
GPR1 RT-PCR	GPR1_cF_28	AGTGCTGTGGTTCCGACTGAATT
	GPR1_cR_668	CAGAGTTCATATCGACGATCCAA
APT1 RT-PCR reference	APT1_F_456	GTTGAATGTGCTTGCG
	APT1_R_804	CTTTAGCCCCTGTTGG

Introduced restriction enzyme sites are underlined.
